# The Effects of Oral and Enteric *Campylobacter concisus* Strains on Expression of TLR4, MD-2, TLR2, TLR5 and COX-2 in HT-29 Cells

**DOI:** 10.1371/journal.pone.0056888

**Published:** 2013-02-20

**Authors:** Yazan Ismail, Hoyul Lee, Stephen M. Riordan, Michael C. Grimm, Li Zhang

**Affiliations:** 1 School of Biotechnology and Biomolecular Sciences, University of New South Wales, Sydney, New South Whales, Australia; 2 Gastrointestinal and Liver Unit, The Prince of Wales Hospital, Sydney, New South Whales, Australia; 3 Faculty of Medicine, University of New South Wales, Sydney, New South Whales, Australia; 4 St George Clinical School, University of New South Wales, Sydney, New South Whales, Australia; Charité, Campus Benjamin Franklin, Germany

## Abstract

*Campylobacter concisus*, a Gram-negative bacterium that colonizes the human oral cavity, has been shown to be associated with inflammatory bowel diseases (IBD). The effects of different *C. concisus* strains on intestinal epithelial expression of Toll like receptors (TLR) have not been investigated. This study examined the effects of *C. concisus* strains isolated from patients with IBD and controls on expression of TLR4, its co-receptor myeloid differentiation factor (MD)-2; TLR2, TLR5, cyclooxygenase-2 (COX-2) and interleukin (IL)-8 in HT-29 cells.

Fourteen oral and enteric *C. concisus* strains isolated from patients with IBD and healthy controls were co-incubated with HT-29 cells. Expression of TLR4, MD-2, TLR2, TLR5 and COX-2 in HT-29 cells in response to *C. concisus* infection was examined by Western blot, flow cytometry analysis and immunofluorescent staining visualized by confocal microscope. Production of IL-8 was evaluated by enzyme-linked immunosorbent assay.

Both oral and enteric *C. concisus* strains upregulated expression of TLR4 in HT-29 cells. The levels of glycosylated TLR4 (Gly-TLR4) and surface TLR4 induced by *C. concisus* strains isolated from patients with IBD were significantly higher than those induced by *C. concisus* strains isolated from the healthy controls. Four *C. concisus* strains isolated from patients with IBD induced more than two-fold increase of surface expression of MD-2. *C. concisus* did not affect expression of TLR2 and TLR5. All *C. concisus* strains induced production of IL-8 and COX-2 in HT-29 cells.

This study shows that some *C. concisus* strains, most from patients with IBD, upregulate surface expression of TLR4 and MD-2 in HT-29 cells. These data suggest that a potential role of specific *C. concisus* strains in modulating the intestinal epithelial responses to bacterial LPS needs to be investigated.

## Introduction

*Campylobacter concisus* is a Gram-negative bacterium commonly found in the human oral cavity [1], [2]. *C. concisus* is motile by means of a single polar flagellum and requires H_2_-enriched microaerobic conditions for growth [3].

*C. concisus* has been shown to be associated with inflammatory bowel disease (IBD) [4], [5], [6], [7], [8]. A significantly higher prevalence of *C. concisus* in intestinal biopsies and fecal samples of patients with IBD was detected as compared with controls [4], [5], [6], [7]. IBD is a chronic inflammatory disorder of the gastrointestinal tract; its most common incidence is in adolescents and young adults [9], [10]. The two major types of IBD are Crohn's disease (CD) and ulcerative colitis (UC). The aetiology of IBD is unknown. Multiple factors including intestinal microbiota, genetic factors, environmental factors and aberrant responses in the innate and adaptive immune system contribute to the development of IBD [9], [10].

The intestinal microbiota play a key role in the development of IBD. Studies from both human and animal models of IBD have demonstrated that colitis does not occur in the absence of intestinal microbiota [11], [12], [13], [14]. Furthermore, a breakdown in tolerance of the gut immune system to commensal intestinal bacteria in patients with IBD has been detected [15], [16]. Despite the strong evidence supporting the important role of intestinal microbiota in the development of IBD, a causative agent(s) of human IBD remains elusive.

*C. concisus* colonizing the oral cavity has been shown to be a source of *C. concisus* colonizing the intestinal tissues in some patients with IBD [17]. Recently *C. concisus* was detected in fecal and saliva samples of domestic dogs and cats [18], [19]. This bacterium has also been isolated from chicken and beef meat [20]. These data suggest that domestic pets, chicken and beef meat may also serve as a source of human intestinal colonization of *C. concisus.*

Despite its high prevalence in the intestinal tract of patients with IBD, whether *C. concisus* contributes to the pathogenesis of IBD is unknown. A number of studies have examined the effects of *C. concisus* on intestinal epithelial cells using *in vitro* cell culture models. Some oral and enteric *C. concisus* strains were shown to be invasive to Caco2 cells [5], [17]. Increased intestinal epithelial permeability and epithelial apoptosis were also observed following the incubation of Caco2 cells with both oral and enteric *C. concisus* strains [5], [21]. Enteric *C. concisus* strains were shown to induce the production of IL-8 in HT-29 cells [5], [22]. These data suggest that some *C. concisus* strains have a potential to cause enteric diseases.

The effects of *C. concisus* strains on intestinal epithelial expression of Toll like receptors (TLR) have not been investigated. A low level expression of TLR4 and its co-receptor myeloid differentiation factor (MD)-2 in intestinal epithelial cells under normal physiological conditions is a strategy of the intestinal immune system to avoid dysregulated inflammatory responses to bacterial lipopolysaccharide (LPS) [23]. In patients with IBD, increased levels of intestinal expression of TLR4 and other proinflammatory molecules such as cyclooxygenase-2 (COX-2) and interleukin (IL)-8 have been observed [24], [25]. Examination of the effects of different *C. concisus* strains on intestinal epithelial expression of TLRs and other proinflamatory molecules will further shed light on whether some *C. concisus* strains have the potential to contribute to the pathogenesis of human enteric diseases including IBD. Given that *C. concisus* is a Gram-negative flagellated bacterium, in this study, we examined the effects of both oral and enteric *C. concisus* strains isolated from patients with IBD and controls on intestinal epithelial expression of TLR4 and its co-receptor myeloid differentiation factor (MD)-2, which recognizes LPS found in Gram-negative bacteria, of TLR2, which recognizes bacterial lipoproteins, and of TLR5, which recognizes bacterial flagellin. Furthermore, the induction of COX-2 and IL-8 in HT-29 cells by both oral and enteric *C. concisus* strain was assessed.

## Results

### Expression of TLR4, MD-2, TLR2, TLR5 and COX-2 in HT-29 cells induced by different *C. concisus* strains detected by Western blot

**Figure 1:**
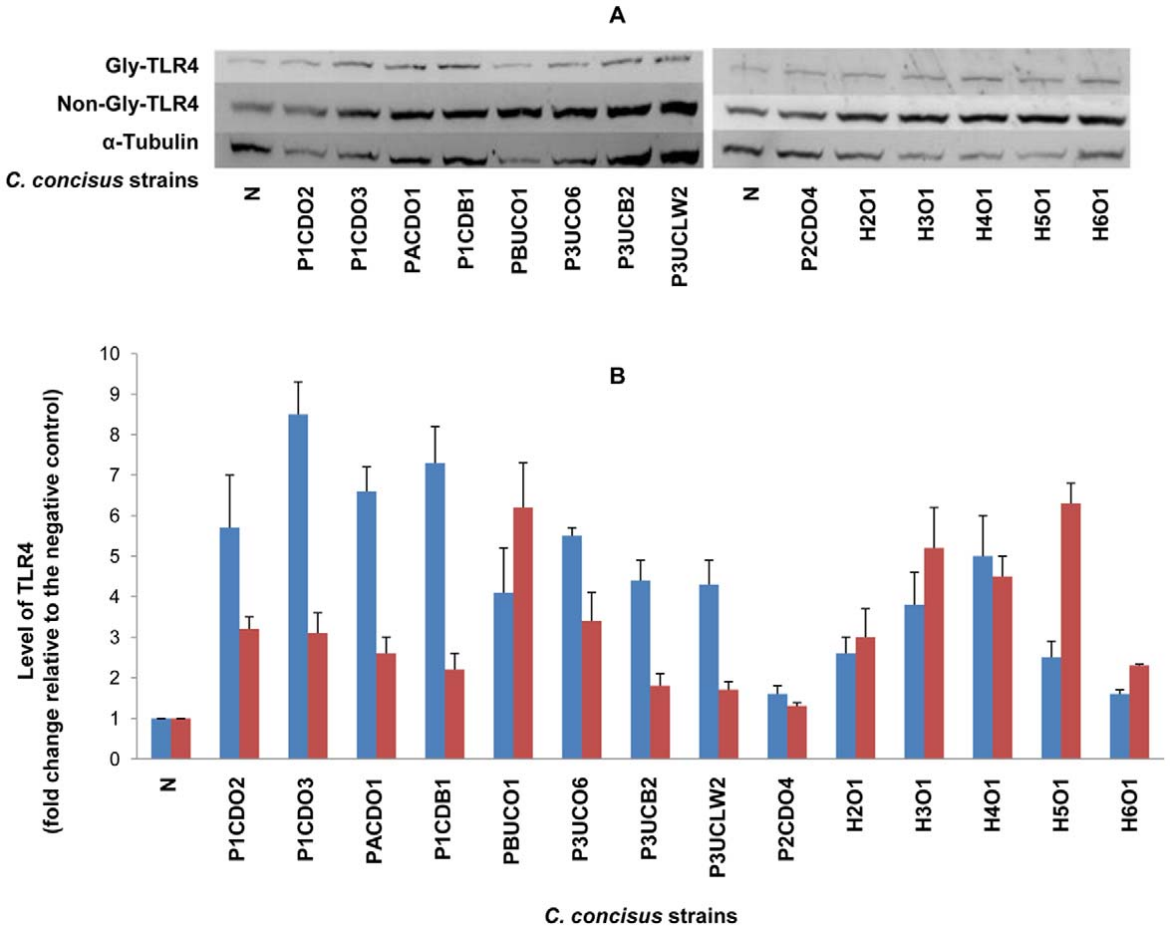
Detection of TLR4 by Western blot in HT-29 cells infected with *C. concisus* strains. HT-29 cells were lysed following incubation with *C. concisus* strains for 24 hours. Expression of TLR4 in HT-29 cells was detected by Western blot. Two bands, the Glycosylated TLR 4 (Gly-TLR4) and non-glycosylated TLR4 (Non-Gly-TLR4), were revealed on Western blot. The levels of Gly-TLR4 and Non-Gly-TLR4 of each sample were expressed as the fold change of the band intensity relative to the band intensity of the negative control (HT-29 cells without *C. concisus* infection), after normalization to the intensity of the internal control α-Tubulin of the same sample. A: Representative Western blot of Gly-TLR4 (120 kD), Non-Gly-TLR4 (95 kD) and α-Tubulin (55 kD). B: Level of Gly-TLR4 (blue column) and Non-Gly-TLR4 (red column) induced by different *C. concisus* strains; data were the average of triplicate experiments ± standard error. N: negative control. H2O1-H6O1: *C. concisus* strains isolated from healthy controls. The remaining nine *C. concisus* strains were from patients with IBD. The average level of Gly-TLR4 induced by *C. concisus* strains from patients with IBD was significantly higher than that induced by *C. concisus* strains from healthy controls (*P*<0.05).

**Figure 2:**
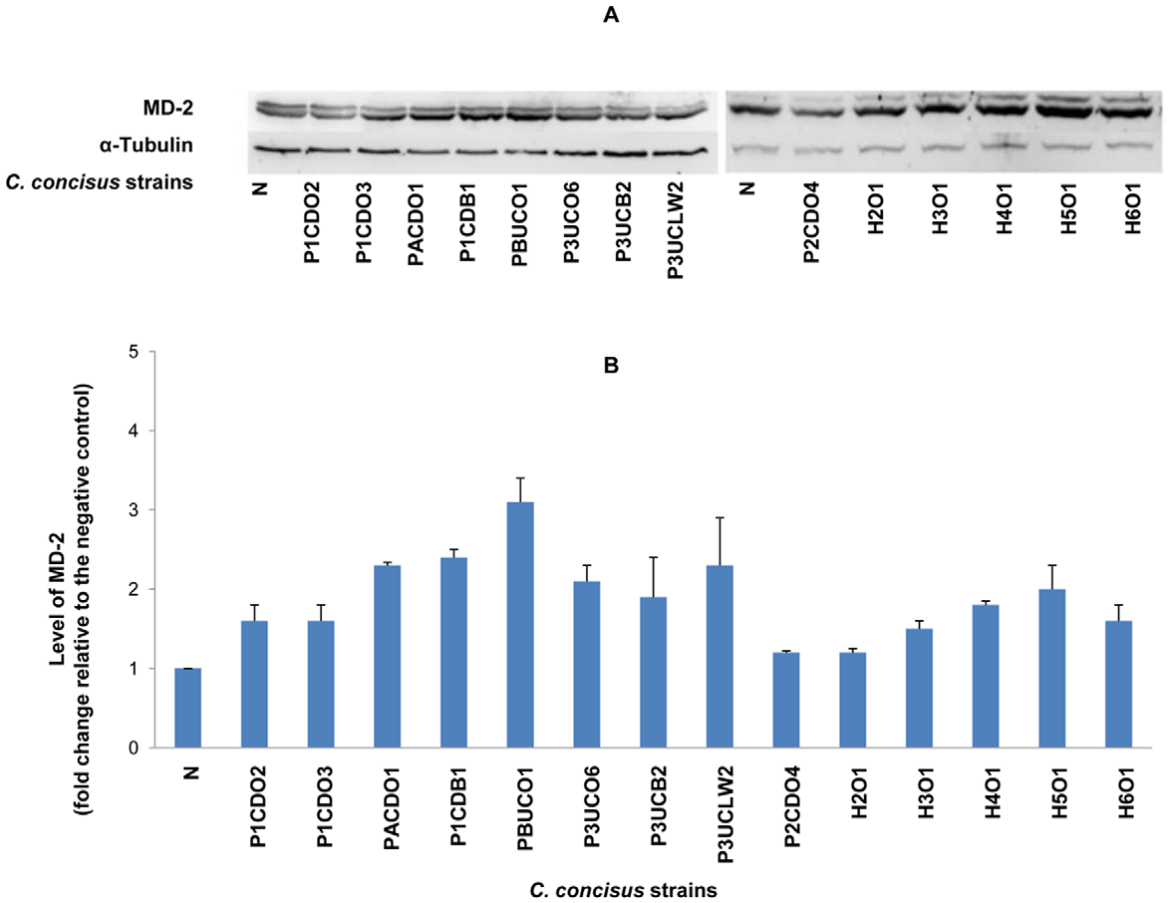
Detection of MD-2 by Western blot in HT-29 cells infected with *C. concisus* strains. HT-29 cells were lysed following incubation with *C. concisus* strains for 24 hours. Expression of MD-2 in HT-29 cells was detected by Western blot. Two bands, the Glycosylated MD-2 and non-glycosylated MD-2, were revealed on Western blot. Giving the close distance of Glycosylated MD-2 and non-glycosylated MD-2 protein bands which made it difficult to analyze the bands separately, these two protein bands were analyzed together. The level of MD-2 was expressed as the fold change of the normalized band intensity of a sample relative to the normalized band intensity of the negative control (HT-29 cells without *C. concisus* infection), after normalization to the intensity of the internal control α-Tubulin of the same sample. A: Representative Western blot of MD-2 (23–25 kD) and α-Tubulin (55 kD). B: Level of total MD-2 induced by different *C. concisus* strains; data were the average of triplicate experiments ± standard error. N: negative control. H2O1-H6O1: *C. concisus* strains isolated from healthy controls. The remaining nine *C. concisus* strains were from patients with IBD. The average level of MD-2 induced by *C. concisus* strains from patients with IBD was not significantly higher than that induced by *C. concisus* strains from healthy controls (*P*>0.05).

**Figure 3:**
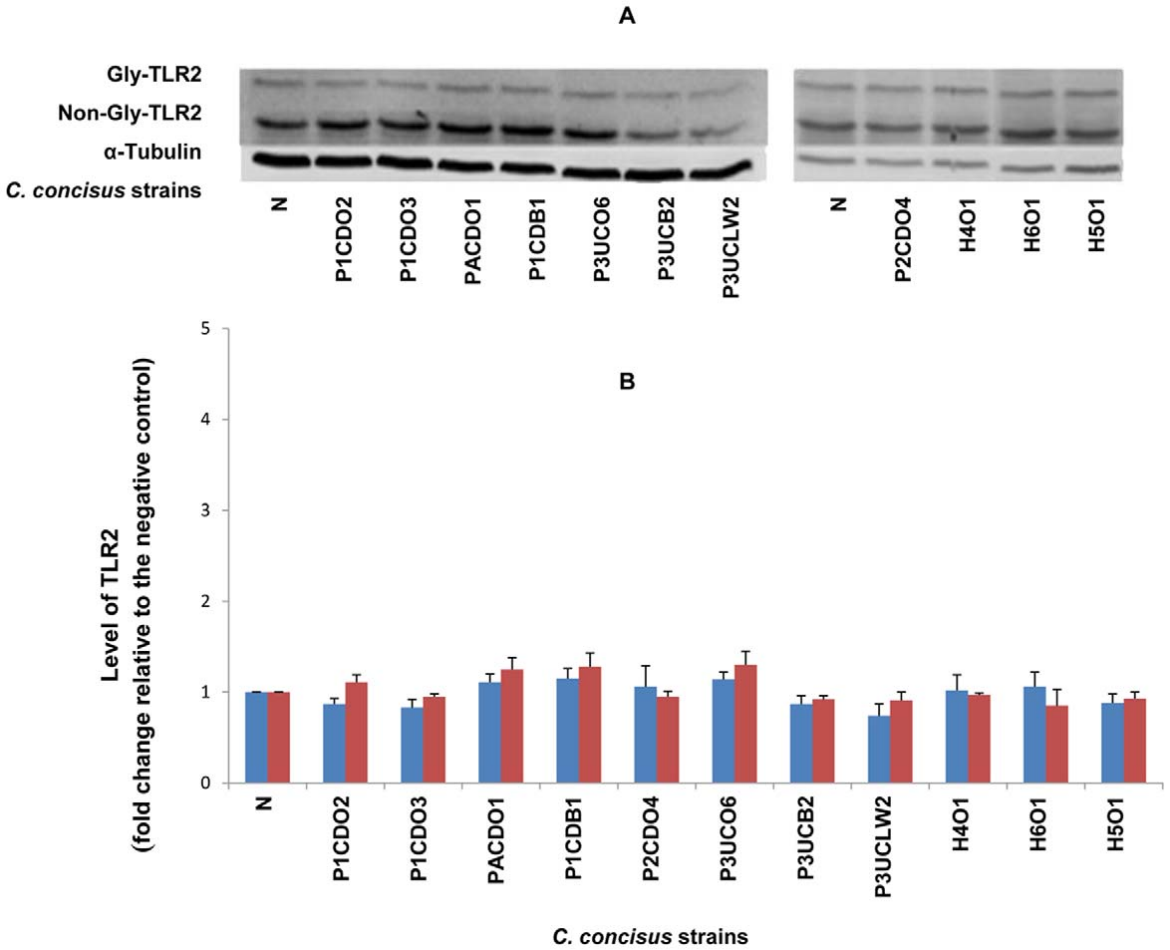
Detection of TLR2 by Western blot in HT-29 cells infected with *C. concisus* strains. HT-29 cells were lysed following incubation with *C. concisus* strains for 24 hours. Expression of TLR2 in HT-29 cells was detected by Western blot. Two bands, the Glycosylated TLR 2 (Gly-TLR4) and non-glycosylated TLR2 (Non-Gly-TLR4), were revealed on Western blot. The levels of Gly-TLR2 and Non-Gly-TLR2 of each sample were expressed as the fold change of the band intensity relative to the band intensity of the negative control (HT-29 cells without *C. concisus* infection), after normalization to the intensity of the internal control α-Tubulin of the same sample. A: Representative Western blot of Gly-TLR2 (100 kD), Non-Gly-TLR2 (90 kD) and α-Tubulin (55 kD). B: Level of Gly-TLR2 (blue column) and Non-Gly-TLR2 (red column) induced by different *C. concisus* strains; data were the average of triplicate experiments ± standard error. N: negative control. H2O1-H6O1: *C. concisus* strains isolated from healthy controls. The remaining *C. concisus* strains were from patients with IBD. The average levels of Gly-TLR2 and Non-Gly-TLR2 induced by *C. concisus* strains from patients with IBD were not significantly different that induced by *C. concisus* strains from healthy controls (*P*>0.05).

**Figure 4:**
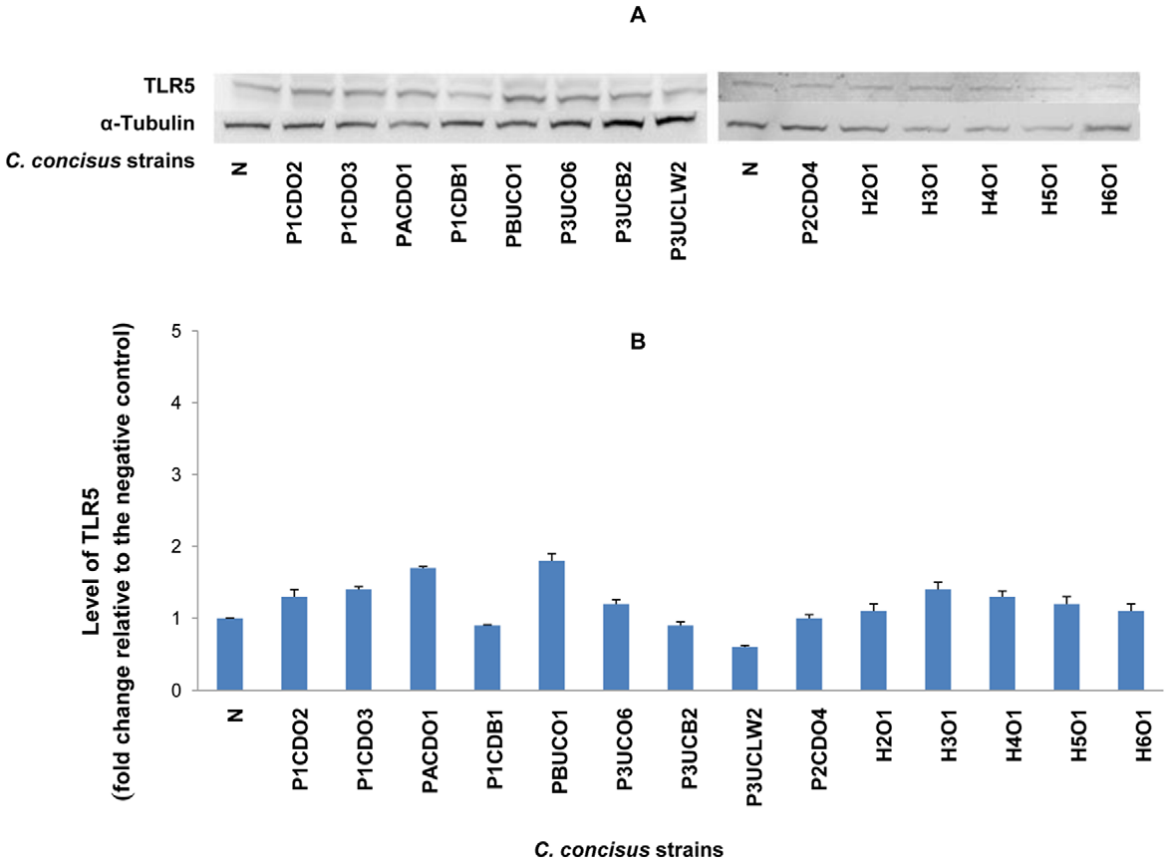
Detection of TLR5 by Western blot in HT-29 cells infected with *C. concisus* strains. HT-29 cells were lysed following incubation with *C. concisus* strains for 24 hours. Expression of TLR5 in HT-29 cells was detected by Western blot. One TLR 5 band was revealed on Western blot. The intensity of TLR5 of each sample was normalized to the intensity of the internal control α-Tubulin of the same sample. The level of TLR5 was expressed as the fold change of the normalized band intensity of a sample relative to the normalized band intensity of the negative control (HT-29 cells without *C. concisus*). A: Representative Western blot of TLR5 (110 kD) and α-Tubulin (55 kD). B: Levels of TLR5 induced by different *C. concisus* strains; data were the average of triplicate experiments ± standard error. N: negative control. H2O1-H6O2: *C. concisus* strains isolated from healthy controls. The remaining nine *C. concisus* strains were from patients with IBD. The average level of TLR5 induced by *C. concisus* strains from patients with IBD was not significantly higher than that induced by *C. concisus* strains from healthy controls (*P*>0.05).

**Figure 5:**
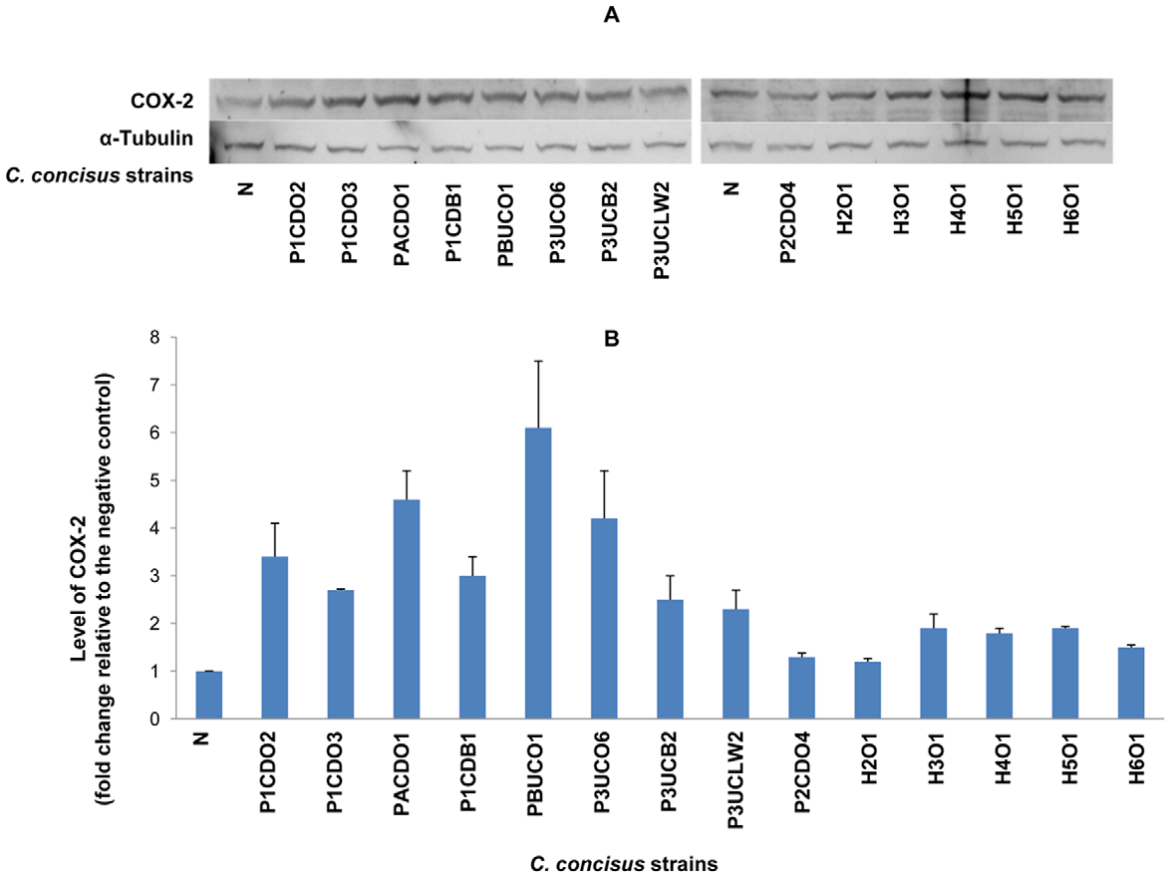
Detection of COX-2 by Western blot in HT-29 cells infected with *C. concisus* strains. HT-cells 29 were lysed following incubation with *C. concisus* strains for 24 hours. Expression of COX-2 in HT-29 cells was detected by Western blot. The intensity of COX-2 band of each sample was normalized to the intensity of the internal control α-Tubulin of the same sample. The level of COX-2 was expressed as the fold change of the normalized band intensity of a sample relative to the normalized band intensity of the negative control (HT-29 cells without *C. concisus* infection). A: Representative Western blot of COX-2 (70 kD) and α-Tubulin (55 kD). B: Levels of COX-2 induced by different *C. concisus* strains; data were the average of triplicate experiments ± standard error. N: negative control. H2O1-H6O1: *C. concisus* strains isolated from healthy controls. The remaining nine *C. concisus* strains were from patients with IBD. The average level of COX-2 induced by *C. concisus* strains from patients with IBD was significantly higher than that induced by *C. concisus* strains from healthy controls (*P*<0.05).

On Western blot, TLR4, MD-2 and TLR2 revealed two protein bands (glycosylated and non-glycosylated proteins); TLR5 and COX-2 revealed one protein band. The intensity of each protein band detected by Western blot was normalized to the intensity of α-Tubulin (internal control) of the same sample. The levels of TLR4, MD-2, TLR2, TLR5 and COX-2 in each sample were expressed as the fold change of the normalized band intensity relative to the normalized band intensity of the negative control (HT-29 cells without *C. concisus* infection), which are shown in Table 1. For TLR4 and TLR2, the glycosylated receptors (Gly-TLR4 and Gly-TLR2) and the non-glycosylated receptors (Non-Gly-TLR4 and Non-Gly-TLR2) were analyzed separately. For MD-2, the glycosylated MD-2 and the non-glycosylated MD-2 were analyzed together, due to the narrow distance of glycosylated MD-2 and the non-glycosylated MD-2 bands on Western blot that made it difficult to analyze the two bands separately. The representative Western blot patterns and the schematic levels of TLR4, MD-2, TLR2, TLR5 and COX-2 are shown in Figure 1, Figure 2, Figure 3, Figure 4 and Figure 5 respectively.

**Table 1:** Expression of TLR4, MD-2, TLR2, TLR5 and COX-2 in HT-29 cells induced by *C. concisus* strains detected by Western blot.

	Gly-TLR4	Non-Gly-TLR4	MD-2	Gly-TLR2	Non-Gly-TLR2	TLR5	COX-2
P1CDO2	5.7±1.3	3.2±0.3	1.6±0.2	0.87±0.06	1.11±0.08	1.3±0.1	3.4±0.7
P1CDO3	8.5±0.8	3.1±0.5	1.6±0.2	0.83±0.09	0.95±0.03	1.4±0.04	2.7±0.02
PACDO1	6.6±0.6	2.6±0.4	2.3±0.04	1.11±0.09	1.25±0.13	1.7±0.02	4.6±0.6
P1CDB1	7.3±0.9	2.2±0.4	2.4±0.1	1.15±0.11	1.28±0.15	0.9±0.01	3.0±0.4
PBUCO1	4.1±1.1	6.2±1.1	3.1±0.3	ND	ND	1.8±0.1	6.1±1.4
P3UCO6	5.5±0.4	3.4±0.7	2.1±0.2	1.14±0.08	1.30±0.15	1.2±0.06	4.2±1.0
P3UCB2	4.4±0.5	1.8±0.3	1.9±0.5	0.87±0.09	0.92±0.04	0.9±0.05	2.5±0.5
P3UCLW2	4.3±0.6	1.7±0.2	2.3±0.6	0.74±0.13	0.91±0.09	0.6±0.02	2.3±0.4
P2CDO4	1.6±0.2	1.3±0.08	1.2±0.02	1.06±0.23	0.95±0.06	1.0±0.05	1.3±0.08
H2O1	2.6±0.4	3.0±0.7	1.2±0.05	ND	ND	1.1±0.1	1.2±0.07
H3O1	3.8±0.8	5.2±1.0	1.5±0.1	ND	ND	1.4±0.1	1.9±0.3
H4O1	5.0±1	4.5±0.5	1.8±0.05	1.02±0.17	0.97±0.02	1.3±0.08	1.8±0.1
H5O1	2.5±0.4	6.3±0.5	2.0±0.3	0.88±0.10	0.93±0.07	1.2±0.1	1.9±0.04
H6O1	1.6±0.1	2.3±0.03	1.6±0.2	1.06±0.16	0.85±0.18	1.1±0.1	1.5±0.05

The levels of molecules were expressed as the fold change of the band intensity of HT-29 cells infected with a *C. concisus* strain relative to the band intensity of the negative control (HT-29 cells without *C. concisus* infection), after normalization to the intensity of the internal control α-Tubulin of the same sample. Data were the average of triplicate experiments ± standard error. Gly: glycosylated. Non-Gly: non-glycosylated. Five *C. concisus* strains (H2O1-H6O1) were isolated from healthy controls; the remaining nine strains were from patients with IBD. P1CDB1, P3UCB2 and P3UCLW2 were enteric strains; the remaining 11 strains were oral strains.

*C. concisus* strains obtained from both patients with IBD and controls upregulated the expression of TLR4 in HT-29 cells. The levels of TLR4 induced by different *C. concisus* strains varied. Of the 14 *C. concisus* strains examined, 12 strains induced more than two-fold increase of expression of Gly-TLR4 and 11 strains induced more than two-fold increase of expression of Non-Gly-TLR4 (Figure 1 and Table 1). The average level of Gly-TLR4 induced by the nine *C. concisus* strains isolated from patients with IBD was significantly higher than that induced by the five *C. concisus* strains (H2O1-H6O1) isolated from healthy controls (5.3±0.7 vs 3.1±0.6, *P*<0.05). The average level of Non-Gly-TLR4, induced by *C. concisus* strains from patients with IBD was not statistically different from that induced by the *C. concisus* strains from the healthy controls (2.8±0.5 vs 4.3±0.7, *P*>0.05). The average levels of Gly-TLR4 and Non-GlyTLR4 induced by the five oral *C. concisus* strains isolated from patients with IBD were 5.33±2.33 and 3.31±1.61 respectively, which were not significantly different from that induced by the three enteric *C. concisus* strains isolated from patients with IBD (5.33±1.70 and 1.90±0.27 respectively) (*P*>0.05).

Six *C. concisus* strains induced more than two-fold increase of expression of MD-2; five of these strains were from patients with IBD (Figure 2 and Table 1). The average level of MD-2 induced by *C. concisus* strains isolated from patients with IBD was not statistically different from that induced by *C. concisus* strains isolated from healthy controls (1.92±0.15 vs 1.59±0.21, *P*>0.05). The average level of MD-2 induced by the oral *C. concisus* strains isolated from patients with IBD was not statistically different from that induced by the enteric *C. concisus* strains isolated from patients with IBD (1.77±0.20 vs 2.17±0.14, *P*>0.05).

*C. concisus* strains did not affect the expression of TLR2 and TLR5 in HT-29 cells. The changes of TLR2 and TLR5 expression in HT-29 cells infected with *C. concisus* strains were all below two-fold in comparison to the levels of these two proteins in HT-29 cells without *C. concisus* infection (Figure 3, Figure 4 and Table 1).

Of the 14 *C. concisus* strains examined, eight strains isolated from patients with IBD induced more than two fold increase of expression of COX-2 (Figure 5, Table 1). The average level of COX-2 induced by *C. concisus* strains isolated from patients with IBD was significantly higher than that induced by *C. concisus* strains isolated from healthy controls (3.34±1.43 vs 1.66±0.30, *P*<0.05) (Figure 5, Table 1).

### Expression of TLR4, MD-2, TLR2, TLR5 and COX-2 in HT-29 cells induced by different *C. concisus* strains detected by flow cytometry analysis

For flow cytometry analysis, the levels of surface TLR4, MD-2, TLR2 and TLR5 (non-permeabilized cells) and total TLR4, MD-2, TLR2, TLR5 and COX-2 (permeabilized cells) were expressed as the fold change of the mean channel fluorescence intensity (MFI) of HT-29 cells infected with a *C. concisus* strain relative to the MFI of the non-infected HT-29 cells (HT-29 cells without *C. concisus* infection).

**Figure 6:**
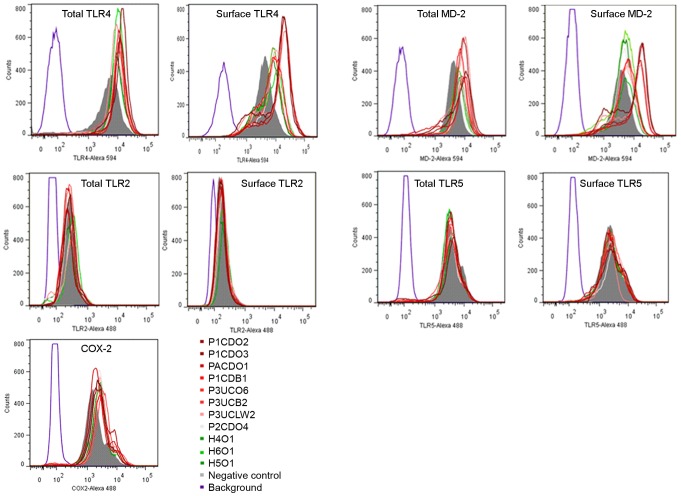
Detection of TLR4, MD-2, TLR2, TLR5 and COX-2 in HT-29 cells by flow cytometry analysis. Flow cytometry histogram showing the expression of surface and total TLR4, MD-2, TLR2, TLR5 and COX-2 in HT-29 cells with and without infection of *C. concisus* strains. A surface expression was measured from non-permeabilized cells and a total expression was measured from permeabilized cells. Background (purple) was from HT-29 cells that were not exposed to antibodies. Negative control (grey): HT-29 cells without *C. concisus* infection. H4O1, H5O1 and H6O1 were strains isolated from healthy controls (green); the remaining eight *C. concisus* strains were from patients with IBD (red).

All 11 *C. concisus* strains examined upregulated both surface expression and total expression of TLR4 in HT-29 cells (Figure 6 and Table 2). Nine strains induced more than two fold increase of surface expression of TLR4 (Table 2). The average level of surface TLR4 induced by *C. concisus* strains isolated from patients with IBD was significantly higher than that induced by *C. concisus* strains isolated from healthy controls (3.70±0.46 vs 1.93±0.05, *P*<0.05). The average level of total TLR4 induced by *C. concisus* strains isolated from patients with IBD was not statistically different from that induced by the *C. concisus* strains from the healthy controls (1.81±0.08 vs 1.63±0.05, *P*>0.05).

**Table 2:** Expression of TLR4, MD-2, TLR2, TLR5 and COX-2 in HT-29 cells induced by *C. concisus* strains detected by flow cytometry.

Strain	Surface TLR4	Total TLR4	Surface MD-2	Total MD-2	Surface TLR2	Total TLR2	Surface TLR5	Total TLR5	COX-2
P1CDO2	4.18±0.56	2.10±0.19	2.46±0.32	1.87±0.07	1.11±0.16	0.99±0.02	1.11±0.06	1.16±0.11	1.43±0.08
P1CDO3	5.03±0.67	1.92±0.18	2.96±0.39	1.86±0.07	0.99±0.07	0.87±0.05	0.99±0.03	1.07±0.05	1.38±0.08
PACDO1	5.40±0.71	1.74±0.15	2.66±0.30	1.49±0.09	1.12±0.16	0.97±0.02	1.01±0.06	1.12±0.09	1.38±0.09
P1CDB1	3.50±0.26	2.12±0.09	1.90±0.18	1.69±0.08	1.13±0.14	0.94±0.04	0.91±0.12	1.08±0.05	1.53±0.04
P2CDO4	2.30±0.29	1.65±0.09	1.54±0.15	1.91±0.10	1.19±0.14	1.14±0.04	1.14±0.10	0.97±0.05	1.33±0.02
P3UCO6	2.23±0.22	1.62±0.11	1.51±0.04	2.00±0.17	1.32±0.33	1.28±0.24	1.13±0.03	1.06±0.05	1.58±0.13
P3UCB2	4.61±0.61	1.51±0.11	2.29±0.29	1.77±0.04	1.21±0.11	1.01±0.11	0.73±0.09	0.85±0.19	1.33±0.08
P3UCLW2	2.30±0.23	1.82±0.07	1.46±0.03	1.61±0.06	1.24±0.12	1.10±0.05	1.10±0.13	1.08±0.05	1.44±0.06
H4O1	1.84±0.20	1.64±0.08	1.36±0.06	1.68±0.10	1.20±0.19	0.95±0.04	1.02±0.06	0.97±0.04	1.45±0.03
H5O1	1.92±0.21	1.54±0.05	1.40±0.07	1.76±0.10	1.36±0.13	1.05±0.06	1.00±0.03	1.24±0.10	1.27±0.05
H6O1	2.02±0.23	1.71±0.09	1.32±0.05	1.40±0.07	1.14±0.11	1.17±0.04	1.02±0.05	0.96±0.04	1.35±0.07

The levels of molecules were expressed as the fold change of the mean channel fluorescence intensity (MFI) of HT-29 cells infected with a *C. concisus* strain relative to the MFI of the non-infected HT-29 cells (HT-29 cells without *C. concisus* infection). A surface expression was measured from non-permeabilized cells and a total expression was measured from permeabilized cells. Data were the average of triplicate experiments ± standard error. Three *C. concisus* strains (H4O1, H5O1 and H6O1) were isolated from healthy controls; the remaining eight strains were from patients with IBD. P1CDB1, P3UCB2 and P3UCLW2 were enteric strains; the remaining 11 strains were oral strains.

Four *C. concisus* strains isolated from patients with IBD, P1CDO2, P1CDO3, PACDO1 and P3UCB2, induced more than two-fold increase of surface expression of MD-2 (Figure 6 and Table 2). The average level of surface MD-2 induced by *C. concisus* strains isolated from patients with IBD was higher than that induced by *C. concisus* strains isolated from healthy controls (2.10±0.57 vs 1.36±0.04). However, this was not statistically significant (*P* = 0.059). The average level of total MD-2 induced by *C. concisus* strains isolated from patients with IBD was not statistically different from that induced by *C. concisus* strains isolated from healthy controls (1.78±0.17 vs 1.61±0.19, *P*>0.05).

*C. concisus* strains did not affect the expression of TLR2 and TLR5 (Figure 6 and Table 2) in HT-29 cells.

All *C. concisus* strains induced an increased expression of COX-2 (Figure 6). However, the increment levels were all below two-fold (Table 2).

### Confocal microscopy observation of TLR4, MD-2, TLR2, TLR5 and COX-2 in HT-29 cells

**Figure 7:**
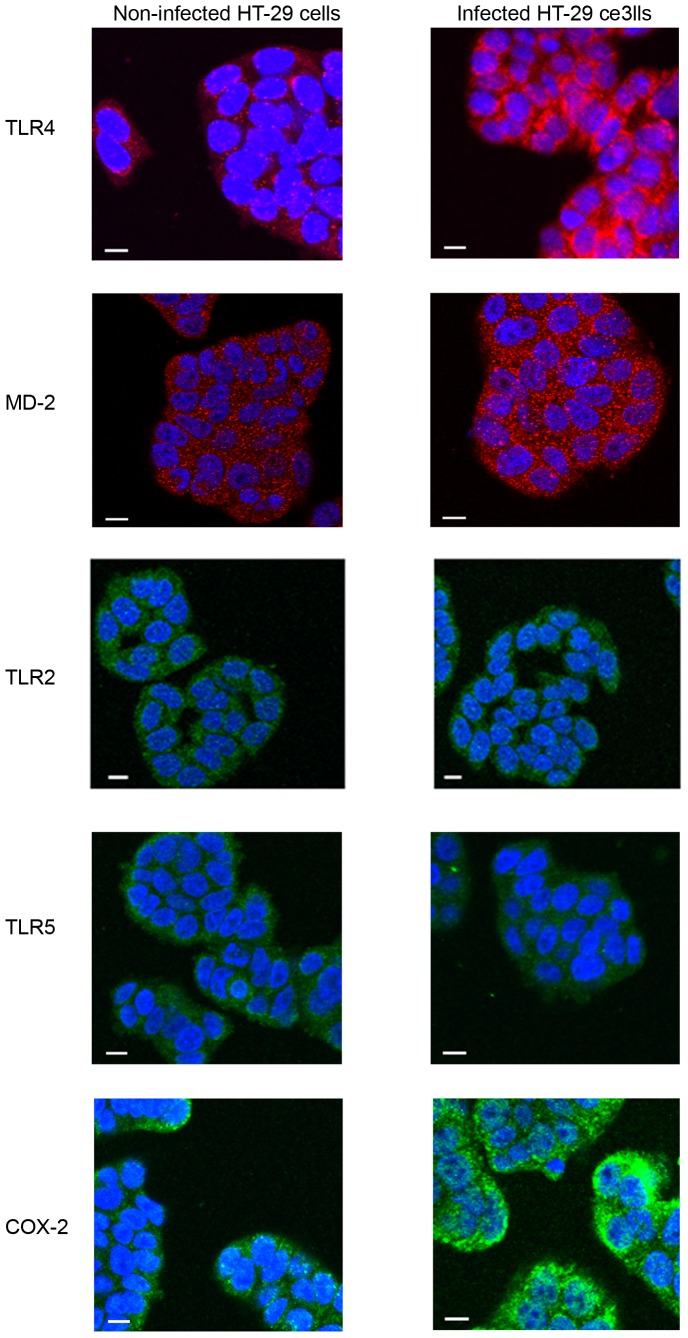
Confocal microscope image of TLR4, MD-2, TLR2, TLR5 and COX-2 expression in HT-29 cells with and without *C. concisu* infection. HT-29 cells were incubated with a representative *C. concisus* strain (P1CDB1) for 24 hours. Expression of TLR4, MD-2, TLR2, TLR5 and COX-2 in HT-29 cells with and without *C. concisus* infection were detected by immunostained using specific antibodies, followed by Alex Fluor conjugated secondary antibodies. The image was viewed using an Olympus FluoView FV1000 Confocal laser scanning microscope. The secondary antibodies used for detection of TLR4 and MD-2 were conjugated with Alexa Fluor 594 (emission colour red). The secondary antibodies used for detection of TLR2, TLR5 and COX-2 were conjugated with Alexa Fluor 488 (emission colour green). Scale Bar = 10 µm.

The effects of *C. concisus* strains on expression of TLR4, MD-2, TLR2, TLR5 and COX-2 in HT-29 cells were visualized by confocal microscopy analysis. Confocal microscopy images, which showed an increased expression of TLR4 and COX-2, a slightly increased expression of MD-2 and no change of TLR5 and TLR2 in HT-29 after coincubation with a representative *C. concisus* strain (P1CDB1) for 24 hours are shown in Figure 7.

### IL-8 production in HT-29 cells induced by oral and enteric *C. concisus* strains

**Figure 8:**
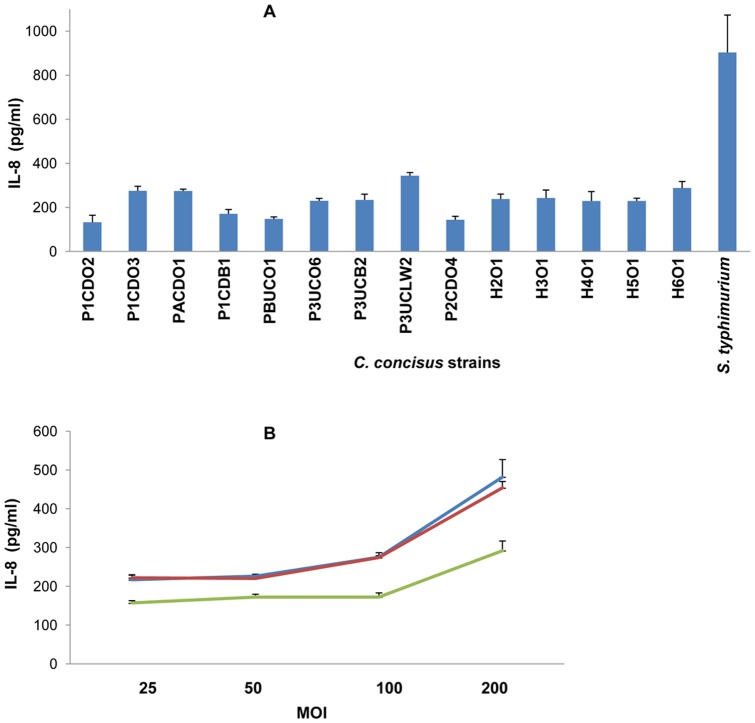
Production of IL-8 by HT-29 cells induced by *C. concisus* strains. **A.** HT-29 cells were incubated with *C. concisus* strains at a multiplicity of infection (MOI) of 100 for 24 hours. Concentrations of IL-8 in the cell culture supernatants were measured by ELISA. The basal production of IL-8 (HT-29 cells without *C. concisus* infection) has been subtracted from values shown in Figure 8A. Data were the average of triplicates ± standard error. The level of IL-8 induced by *C. concisus* strains from patients with IBD was not statistically different from that induced by *C. concisus* strains from the healthy controls (*P*>0.05). Five *C. concisus* strains (H2O1-H6O1) were isolated from healthy controls; the remaining nine strains were from patients with IBD. **B.** HT-29 cells were incubated with three *C. concisus* strains respectively at four different MOIs (25, 50, 100 and 200) for 24 hours. The three *C. concisus* strains used were P1CDO2 (blue), P1CDO3 (red) and P1CDB1 (green). Concentrations of IL-8 in the cell culture supernatants were measured by ELISA. The basal production of IL-8 (HT-29 cells without *C. concisus* infection) has been subtracted from values shown in Figure 8B. Data were the average of triplicates ± standard error.

The concentrations of IL-8 in HT-29 cell culture supernatants were determined by enzyme linked immunosorbent assay (ELISA). The basal amount of IL-8 production by HT-29 cells (HT-29 cells without *C. concisus* infection) was subtracted from the concentration of IL-8 in each sample (HT-29 cells incubated with a *C. concisus* strain) and the results are shown in Figure 8A. The concentration of IL-8 induced by the positive control *Salmonella enterica* serovar Typhimurium LT2 (*Salmonella typhimurium*) was 903±130 pg/ml. Both oral and enteric *C. concisus* strains isolated from patients with IBD and controls induced the production of IL-8. The concentrations (pg/ml) of IL-8 induced by different *C. concisus* strains at a multiplicity of infection (MOI) of 100 were 133±31 for P1CDO2, 276±20 for P1CDO3, 274±9 for PACDO1, 171±19 for P1CDB1, 148±9 for PBUCO1, 234±26 for P3UCO6, 244±14 for P3UCB2, 344±16 for P3UCLW2, 130±11 for P2CDO4, 238±23 for H2O1, 243±36 for H3O1, 229±43 for H4O1, 229±13 for H5O1 and 288±30 for H6O1 (Figure 8 A). The average concentration of IL-8 induced by *C. concisus* strains from patients with IBD was not significantly different from that induced by *C. concisus* strains from healthy controls (217±24 vs 245±11, *P*>0.05).

The relationship between the dose of *C. concisus* and the production of IL-8 in HT-29 cells was assessed by measurement of IL-8 concentrations in cell culture supernatants of HT-29 cells infected with three *C. concisus* strains, P1CDO2, P1CDO3 and P1CDB1, at four different MOIs (25, 50, 100 and 200). Concentrations of IL-8 induced by P1CDO2 *C. concisus* strain at the four MOIs were 217±11, 226±1, 276±12 and 482±45 respectively. Concentrations of IL-8 induced by P1CDO3 *C. concisus* strain at the four MOIs were 222±8, 220±8, 274±5 and 453±16 respectively. Concentrations of IL-8 induced by P1CDB1 *C. concisus* strain at the four MOIs were 157±6, 172±7, 171±11 and 292±25 respectively (Figure 8B).

## Discussion

This study examined the effects of oral and enteric *C. concisus* strains isolated from patients with IBD and controls on intestinal epithelial expression of TLR4, MD-2, TLR2, TLR5, COX-2 and IL-8 using an *in vitro* cell culture model (HT-29 cells).

Functional membrane LPS receptor is a multiple protein complex including at least three proteins, TLR4, MD-2 and CD14 [26]. In physiological conditions, intestinal epithelial cells express a low level of LPS receptor proteins [23], [24], [27], [28]. In patients with IBD, increased intestinal epithelial expression of TLR4 has been detected [24]. In our study, we found that all *C. concisus* strains upregulated the expression of TLR4 in HT-29 cells. Furthermore, we found that most *C. concisus* strains isolated from patients with IBD, rather than those strains isolated from healthy controls, predominately upregulated surface expression of TLR4 and Gly-TLR4 (Figures 1 and 6, Tables 1 and 2). These data suggest that some *C. concisus* strains may have the potential to enhance the intestinal epithelial inflammatory responses to LPS derived from other bacterial species such as those from intestinal commensal bacteria. Further studies are needed to investigate this issue.

TLR4 glycosylation has been shown to be essential in forming the functional LPS receptor [26]. Upregulation of gastric epithelial expression of Gly-TLR4 by the gastric mucosal associated Gram-negative bacterium *Helicobacter pylori* was previously observed. In a study examining the effects of *H. pylori* LC11 and LC20 strains on expression of TLR4 in MKN45 gastric epithelial cells, Su *et al* found that *H. pylori* LC11 strain upregulated the expression of Gly-TLR4 at a much greater level than that induced by the LC20 strain [29]. The difference between these two strains is that LC11 contains the pathogenicity island [30]. The mechanisms by which some *C. concisus* strains more effectively upregulate Gly-TLR4 in HT-29 cells are currently unknown, which remains to be investigated.

Mucosal-associated bacterial species have different effects on TLR4 expression in HT-29 cells. In a study by Furrie *et al*, mRNA levels of TLR4 in HT-29 cells in response to six bacterial species isolated from intestinal biopsies, *Enterococcus faecalis*, *Escherichia coli*, *Peptostreptococcus anaerobium*, *Bifidobacterium longum*, *Bifidobacterium bifidum*, and *Bacteroides fragilis*, were assessed. This study showed that *E. coli* significantly reduced mRNA level of TLR4 and *E. faecalis* significantly increased the mRNA of TLR4 in HT-29 cells. The remaining four bacterial species did not significantly affect the expression of TLR4 in HT-29 cells [31].

MD-2 is a protein that is associated with the extracellular domain of TLR4, which enables TLR4 to function as the LPS receptor [32], [33], [34]. Increased intestinal epithelial expression and sera MD-2 activity in patients with IBD have been reported [35], [36]. In our study, increased MD-2 expression in HT-29 cells following *C. concisus* infection was detected by both Western blot and flow cytometry analysis. However, the levels of increment of MD-2 induced by *C. concisus* were not as great as the increment of TLR4. Some *C. concisus* strains from patients with IBD induced more than two-fold increase of surface expression of MD-2 (Figure 6 and Table 2). Similar to TLR4 glycosylation, MD-2 glycosylation was also shown to be essential in maintaining the functional integrity of LPS receptor [26]. However, in our study we could not assess the levels of glycosylated MD-2 and non-glycosylated MD-2 separately due to the proximity of these two protein bands on Western blot.

TLR5 recognizes a conserved site on bacterial flagellin [37]. The flagellin of *S. typhimurium* is the major epithelial proinflammatory determinant; activating intestinal epithelial expression of TLR5 [38], [39]. However, the flagellin of members of ε-Proteobacteria, which include genera of *Helicobacter*, *Campylobacter* and *Wollinella*, is able to evade recognition by TLR5, owing to altered amino acids at the TLR5 recognition site [40]. In our study *C. concisus* strains showed no or minimal effects on TLR5 expression in HT-29 cells (Figures 4 and 6). Previously we showed that *C. concisus* attached to Caco2 cells using their flagella [41]. The evasion of TLR5 would allow *C. concisus* to attach to the intestinal epithelial cells using flagellum without the notice of the innate immune system, providing the opportunity for this bacterium to modulate the gut innate immune system as discussed above. TLR2 recognizes bacterial lipoproteins. In our study, we found that *C. concisus* did not affect the expression of TLR2 in HT-29 cells.

COX-2 is an inducible enzyme responsible for producing prostaglandins and other important inflammatory mediators [42], [43]. Singer *et al* showed that COX-2 was not detected in normal colonic epithelial cells but was induced in patients with IBD [25]. COX-2 has also been shown to be associated with intestinal epithelial high fluid secretion induced by enteric pathogens [44], [45]. In addition to its involvement in inflammation, COX-2 has been linked to several malignancies including colorectal cancer [46]. In this study, we found that *C. concisus* strains isolated from patients with IBD induced a significantly higher level of COX-2 in HT-29 cells in comparison to *C. concisus* strains isolated from healthy controls by Western blot. However through flow cytometry analysis, a low level of COX-2 increase induced by these *C. concisus* strains was detected. Furthermore, the increased levels of COX-2 induced by *C. concisus* strains isolated from patients with IBD and controls were not significantly different by flow cytometry analysis. As there may be discrepancies in the sensitivities of Western blot and flow cytometry techniques in the detection of low abundance proteins, it is unclear whether this may have an effect on the differing levels of COX-2 detected. Whether the increment of COX-2 induced by *C. concisus* has a biological impact remains to be examined.

The production of IL-8 induced by different *C. concisus* strains in HT-29 cells was investigated. In this study, we did not observe a correlation between the increased expression levels of TLR4 induced by different *C. concisus* strains and the production of IL-8 in HT-29 cells. The reason for this is not clear. One explanation is that in this study, whole bacteria were used in the induction of IL-8 in HT-29 cells. Given that IL-8 is induced by multiple bacterial components through multiple receptors and pathways, it is therefore difficult to correlate its expression levels to a single receptor. The second possibility is that different *C. concisus* strains may have different types of LPS that vary in their abilities to induce proinflammatory mediators, which further complicates the matter of assessing the correlation between the levels of TLR4 and IL-8 between different *C. concisus* strains. This view is supported by many previous studies, as they showed that *C. concisus* is a bacterial species with great diversity [17], [47], [48], [49], [50]. Future studies are required to investigate whether the upregulation of TLR4 by *C. concisus* enhances the intestinal epithelial inflammatory responses to LPS originating from intestinal commensal bacteria, enteric pathogens as well as varying *C. concisus* strains.

Examination of the relationship between *C. concisus* dose and the production of IL-8 in HT-29 cells showed that at lower MOIs (MOI 25–100), an increase in bacterial dose did not affect the production of IL-8. At higher MOIs (MOI 100–200), a bacterial dose dependent production of IL-8 in HT-29 cells was observed (Figure 8B). Interestingly, upregulation of TLR4 in HT-29 cells by *C. concisus* strains does not require a high dose of *C. concisus*. For example, we found that P1CDB1 strain upregulated TLR4 in HT-29 cells at a MOI of 5 and that the upregulation of TLR4 by P1CDB1 at a MOI of 100 was not as efficient as the upregulation of TLR4 by the same strain at a MOI of 25 (data not shown).

In summary, in this study we found that both oral and enteric *C. concisus* strains upregulated expression of TLR4 and MD-2 in HT-29 cells. Some *C. concisus* strains, most of them from patients with IBD, were more effective in regulating surface expression of TLR4 and MD-2, as well as Gly-TLR4. *C. concisus* infection in HT-29 cells did not affect the expression of TLR2 and TLR5. All *C. concisus* strains induced production of IL-8 and COX-2 in HT-29 cells. These data suggest that a potential role of specific *C. concisus* strains in modulating the intestinal epithelial responses to bacterial LPS needs to be investigated.

## Materials and Methods

### Ethics statement

*C. concisus* strains used in this study were isolated in our previous studies [2], [4], [7]. Ethics approvals were granted by the Ethics Committees of the University of New South Wales and the South East Sydney Area Health Service (Ethics Nos: HREC 09237/SESIAHS 09/078, HREC08335/SESIAHS(CHN)07/48 and HREC 06233/SESAHS (ES) 06/164). Written informed consent was obtained from the subjects or the parents/guardians of the minors.

### *C. concisus* strains and cultivation conditions

Fourteen oral (isolated from saliva) and enteric (isolated from intestinal biopsies and feces) *C. concisus* strains we previously isolated were included in this study [2], [4], [7]. Details of the *C. concisus* strains used in this study were listed in Table 3.

**Table 3:** *C. concisus* strains used in this study.

Strain ID	Source	Clinical conditions
P1CDO2	Saliva	Crohn's disease
P1CDO3	Saliva	Crohn's disease
P1CDB1	Intestinal biopsy	Crohn's disease
PACDO1	Saliva	Crohn's disease
P3UCO6	Saliva	Ulcerative colitis
P3UCB2	Intestinal biopsy	Ulcerative colitis
P3UCLW2	Luminal washout	Ulcerative colitis
PBUCO1	Saliva	Ulcerative colitis
P2CDO4	Saliva	Crohn's disease
H2O1	Saliva	Healthy
H3O1	Saliva	Healthy
H4O1	Saliva	Healthy
H5O1	Saliva	Healthy
H6O1	Saliva	Healthy

These *C. concisus* strains were isolated from previous studies [2], [4], [7]. P1CDO2, P1CDO3 and P1CDB1 were isolated from a patient with CD. P3UCO6, P3UCB2 and P3UCLW2 were isolated from a patient with UC. The remaining strains were isolated from individual patients with IBD and healthy controls. P1CDB1 was named as UNSWCD and P1CDB1(UNSWCD) in previous studies [17], [41].

All *C. concisus* isolates were grown in heart infusion broth (Oxoid, Hampshire, UK) containing 2.5% fetal blood serum (FBS) (Bovogen Biologicals, East Keilor, Australia) at 37°C with continuous agitation (120 rpm) under microaerobic condition. The microaerobic condition was generated using *Campylobacter* Gas Generating Kit (Oxoid).

### Cultivation of HT-29 cells

Human intestinal epithelial cell line HT-29 cells (ATCC No. HTB-38), were maintained in McCoy's 5A medium (Invitrogen, California, USA) supplemented with 10% FBS (Bovogen Biologicals), 100 U/ml penicillin and 100 µg/ml streptomycin (Invitrogen). The cells were grown at 37°C in a humidified incubator containing 5% CO2.

### Antibodies used in this study

All antibodies used in this study were purchased from Santa Cruz Biotechnology Inc (Santa Cruz biotechnology Inc, California, USA). Primary antibodies used were polyclonal anti-TLR4 (sc-10741), anti-MD-2 (sc-20668), anti-TLR2 (sc-166900), anti-TLR5 (sc-16243), anti-Cox-2 (sc-1746), and anti-α tubulin (sc-31779). Secondary antibodies conjugated with horseradish peroxidase (HRP) used for Western blot were bovine anti-goat IgG (sc-2352), goat anti-mouse IgG (sc-2031) and goat anti-rabbit IgG (sc-2054). Secondary antibodies used for flow cytometry analysis and immunostaining were Alexa Fluor® 488 donkey anti-goat IgG (A11055), Alexa Fluor® 488 goat anti-mouse IgG (A11029), and Alexa Fluor® 594 goat anti-rabbit IgG (A11037) (Invitrogen).

### Western blot

Expression of TLR4, MD-2, TLR2, TLR5 and COX-2 in response to *C. concisus* infection in HT-29 cells was examined by Western blot. HT-29 cells at a concentration of 5×10^5^ cell/ml were cultured in 6-well cell culture plates (3 ml cell suspension/well). The cells were grown for 48 hours. HT-29 cells were washed five times using Dulbecco's Phosphate-Buffered Saline (D-PBS) and then infected with *C. concisus* at a MOI of 25 in McCoy's 5A medium supplemented with 10% FBS without antibiotics. The cells were further incubated for 24 hours. HT-29 cells without *C. concisus* infection were used as the negative control.

HT-29 cells were harvested from culture plates by scraping and washed with pre-cooled D-PBS. The cells were lysed using RIPA Buffer (50 mM Tris, 150 mM NaCl, 1% Triton X-100, 0.1% SDS) containing a mixture of protease inhibitors (Sigma-Aldrich, Castle Hill Australia). Whole cell lysates were centrifuged twice at 14000 relative centrifugal force (RCF) for 20 minutes at 4°C. Supernatant was collected and stored at −80°C till use. Protein concentrations were determined using Pierce® BCA Protein Assay Kit (Thermo Fisher Scientific, Scoresby, Australia).

*C. concisus* whole cell proteins (30 µg) were separated on 12% sodium dodecyl sulfate (SDS)-polyacrylamide gel under reducing conditions and transferred onto polyvinylidine difluoride (PVDF) membranes for two hour at 100 Volt in a cold room (4°C). PVDF Membrane were immersed in absolute methanol for five seconds then equilibrated in the transfer buffer for 10–20 minutes prior to be used for protein transfer. The transfer buffer consists of 0.3% (w/v) Tris base, 1.44% (w/v) glycine and 20% methanol (v/v). The transfer buffer was pre-chilled on ice prior to use. Following protein transfer, PVDF membranes were blocked with 5% skim milk in washing buffer (0.05% Tween-20 in phosphate buffered saline (PBS)) for two hours then probed with primary antibodies (1∶350) overnight at 4°C, followed by secondary antibodies conjugated with HRP (1∶2500) for 90 minutes at room temperature (RT). Antibodies were diluted using blocking solution. The HRP-labeled antibodies were detected using Immun-Star™ WesterC™ Chemiluminescence Kits (Bio-Rad laboratory, Gladesville, Australia) and a LAS-3000 imaging system (Fujifilm). The intensities of protein bands were analyzed using Image J software (National institute of health, US). Western blot experiments were repeated three times. PVDF membranes were incubated with antibodies for the molecules examined (TLR4, MD-2, TLR2, TLR5 and COX-2) first, then stripped and re-incubacted with anti-alpha-tubulin antibody. One PVDF membrane was used for detection of one of the molecules examined.

### Flow cytometry analysis

Expression of TLR4, MD-2, TLR2, TLR5 and COX-2 in HT-29 cells was detected by flow cytometry analysis. HT-29 cells (10 ml at 5×10^5^ cell/ml) were seeded onto T-25 tissue culture flasks (Nunc, Roskilde, Denmark) for 24 hours, then infected with *C. concisus* at a MOI of 25 and further incubated for 24 hours. HT-29 cells without *C. concisus* infection were used as the negative control.

Following infection with *C. concisus*, HT-29 cells were washed twice with D-PBS and detached from culture flasks by incubating with 0.25% trypsin (Invitrogen) for five minutes (1 ml each flask), followed by deactivation of trypsin using McCoy's 5A medium supplemented with 10% FBS without antibiotics. The cells were washed twice with pre-cooled D-PBS by centrifugation at 300 RCF for 5 minutes at 4°C.

HT-29 cells were fixed for 12 minutes in 3.7% paraformaldehyde in PBS, then permeabilized for 10 minutes with 0.1% Triton X-100 in PBS when required to analyse the total expression of the target protein in cells. HT-29 cells that were not permeabilized were used to analyse the surface expression of the target proteins. Cells were then washed twice using blocking solution (1% Bovine Serum albumin in PBS), and incubated with the blocking solution for 30 minutes at RT, HT-29 cells were then sequentially incubated at RT with a primary antibody (1∶40) and an Alexa Fluor conjugated secondary antibody (5 µg/ml) for one hour and 45 minutes respectively. Primary and secondary antibodies were diluted by blocking solution. Cells were washed three times with the blocking solution between incubations for 10 minutes each time. Cells were collected after each wash by centrifugation at 300 RCF for 5 minutes at 4°C. HT-29 cells that were not exposed to antibodies were used to assess the background signal. Data were acquired by BD LSRFortessa™ SORP cell analyser (BD Biosciences, San Jose, USA) and analysed in Flow Jo software (Tree star inc, OR, US). Flow cytometry experiments were in triplicate and repeated at least twice.

### Immunofluorescence staining and confocal microscopy

Expression of TLR4, MD-2, TLR2, TLR5 and COX-2 in responses to *C. concisus* infection in HT-29 was visualized using immunofluorescence staining and confocal microscopy

HT-29 cells were seeded onto sterile cover-slips placed in 6-well cell culture plates (3 ml of cells at a concentration of 1×10^5^ cell/ml) and allowed to grow for 48 hours. HT-29 cells were then infected with *C. concisus* at a MOI of 25 and incubated for further 24 hours. HT-29 cells without infection of *C. concisus* were used as negative control.

HT-29 cells grown on cover-slips were fixed in 3.7% paraformaldehyde (was diluted by PBS) for 17 minute at RT, permeabilized with 0.1% Triton X-100 in PBS for 10 minutes at RT. The cover-slips carrying HT-29 cells were blocked with 1% bovine serum albumin (Invitrogen) in PBS for one hour at RT. HT-29 cells were then sequentially incubated with a primary antibody (1∶40) and an Alexa Fluor conjugated secondary antibody (5 µg/ml) for 75 minutes respectively, followed by staining with Hoechst 33342 (1.5 µg/ml) (Invitrogen) diluted in PBS for 12 minutes at RT. Both primary and secondary antibodies were diluted in blocking solution. The incubations with the secondary antibodies and the Hoechst 33342 were carried out in the dark at RT.

The cover slips were washed three times with PBS between incubations for 10 minutes at RT. The cover slips were mounted using AF1 antifadent (Citifluor Ltd, London, UK) and HT-29 cells were observed using an Olympus FluoView FV1000 Confocal laser scanning microscope.

### Measurement of IL-8 in HT-29 cell culture supernatant by ELISA

To examine the production of IL-8 by HT-29 cells induced by *C. concisus*, HT-29 cells (5×10^5^cell/ml) were seeded in 24-well cell culture plates (1 ml/each well) and incubated for 48 hours. HT-29 cells were then infected with *C. concisus* and further incubated for 24 hours. Supernatants were collected and centrifuged twice for 2 minutes at 10,000 g. IL-8 secreted in the supernatants was measured by ELISA using Human IL-8 CytoSet™ (Invitrogen) according to manufacturer's instructions.

Supernatant collected from HT-29 cells co-incubated with *S. typhimurium* (UNSW culture collection) and supernatant from HT-29 cells without bacterial infection were used as the positive and negative control respectively.

### Statistic analysis

Data were analyzed by means of unpaired *t* test using GraphPad Prism version 5.1 (San Diego, CA). P-values below 0.05 (two tailed, 95% confidence interval) were considered significant.
